# New Compounds Induce Brassinosteroid Deficient-like Phenotypes in Rice

**DOI:** 10.3390/plants2030521

**Published:** 2013-08-13

**Authors:** Tadashi Matsumoto, Kazuhiro Yamada, Ikuko Iwasaki, Yuko Yoshizawa, Keimei Oh

**Affiliations:** Department of Biotechnology, Faculty of Bioresource Sciences, Akita Prefectural University, 241-438, Shimoshinjo Nakano, Akita 010-0195, Japan

**Keywords:** brassinosteroid biosynthesis inhibitor, plant hormone, azole derivatives

## Abstract

Brassinosteroids (BRs) are steroidal plant hormones with potent plant growth promoting activity. Because BR-deficient mutants of rice exhibit altered plant architecture and important agronomic traits, we conducted a systemic search for specific inhibitors of BR biosynthesis to manipulate the BR levels in plant tissues. Although previous studies have been conducted with BR biosynthesis inhibitors in dicots, little is known regarding the effects of BR biosynthesis inhibition in monocot plants. In this work, we used potent inhibitors of BR biosynthesis in Arabidopsis, and we performed a hydroponic culture of rice seedlings to evaluate the effects of BR biosynthesis inhibition. Among the test compounds, we found that 1-[[2-(4-Chlorophenyl)-4-(phenoxymethyl)-1,3-dioxolan-2-yl]methyl]-1*H*-1,2,4-triazole (**1**) is a potent inhibitor that could induce phenotypes in rice seedlings that were similar to those observed in brassinosteroid deficient plants. The IC_50_ value for the retardation of plant growth in rice seedlings was approximately 1.27 ± 0.43 μM. The IC_50_ value for reducing the bending angle of the lamina joint was approximately 0.55 ± 0.15 μM.

## 1. Introduction

Plant growth and development are regulated by a complex signal transduction mechanism. Brassinosteroids (BRs) serve as important signal mediators with well-defined functions, including extreme dwarfism, delayed senescence, male sterility, and constitutive photomorphogenesis in the dark [1,2,3]. The use of transgenic techniques to manipulate the endogenous BR content has a remarkable effect on plant growth. Overexpression of *DWARF4*, an enzyme that catalyses a rate-limiting step in BR biosynthesis, enhances plant growth and seed yield in Arabidopsis [4]. Similarly, transgenic rice plants overexpressing a sterol C-22 hydroxylase that catalyses a key step in BR biosynthesis presented increases in the biomass and seed yields [5], and the available evidence indicates that the disruption of BR biosynthesis may be a means to improving biomass production [6]. Because BRs control several important agronomic traits, such as flowering, plant architecture, seed yield, and stress tolerance [7,8], various efforts have been made to control the BR levels in plant tissues.

**Figure 1:**
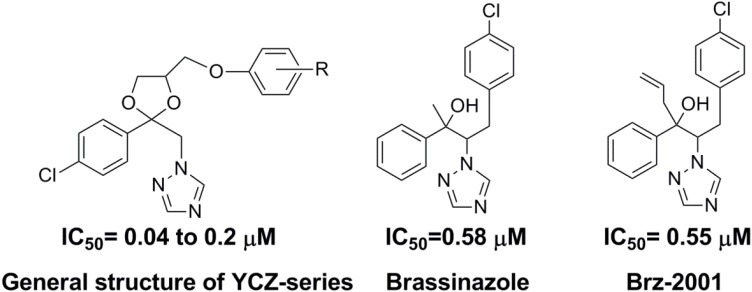
Chemical structures of brassinosteroid biosynthesis inhibitors.

As one of the predominant dietary energy sources for humans, rice is the staple food of over half the world’s population. Great efforts have been made to develop new methods for improving the production and quality of rice. Mutant rice plants that are deficient for BR biosynthesis exhibit dwarf phenotypes with erect leaves [6]. These architectural styles of rice plants are beneficial for dense planting due to the increased light capture for photosynthesis and nitrogen storage for grain filling. Thus, manipulating the BR level in rice plants may be a means to improving rice production. An efficient way to manipulate the BR level in rice plants is the use of specific inhibitors of BR biosynthesis. Indeed, it is believed that the use of specific inhibitors has advantages over genetic mutation because inhibitors can be used at different stages of plant growth and development [9]. Moreover, they can be applied to different plant species with great ease. In this context, searching for selective and potent inhibitors of BR biosynthesis represents a useful approach for dissecting the functions of BRs as well as for regulating plant growth and development. We have conducted a systematic search for specific inhibitors of BR biosynthesis. In the previous work, we reported the discovery of a series of triazole derivatives that exhibit highly selective and potent inhibitory activities on BR biosynthesis in Arabidopsis (designated as the **YCZ-series**; the general structure is shown in Figure 1) [10,11]. Studies on the mechanism of action of this synthetic series on BR biosynthesis suggest that the target sites of the **YCZ-series** are CYP90s, which are responsible for the hydroxylation of the side chain of campesterol, a precursor of BR biosynthesis [12]. 

The discovery of brassinazole was previously reported by Asami *et al.* [13,14], and considerable progress has been made using brassinazole on dicot plants to dissect the functions of BR [9]. However, to the best of our knowledge, there has been no report of potent inhibitors of BR biosynthesis in rice plants. As demonstrated for brassinazole and Brz2001 (the structure is shown in Figure 1), even at a concentration of 10 μM, these inhibitors did not exhibit significant biological activity in rice plants [15]. In contrast, the IC_50_ values of the **YCZ-series** for Arabidopsis were between 0.04 and 0.5 μM [12], and the IC_50_ for brassinazole and Brz-2001 were 0.58 and 0.55 μM [12], respectively. This observation indicates that the potency of the **YCZ-series** as inhibitors of BR biosynthesis in Arabidopsis is stronger than that of brassinazole and/or Brz-2001. Based on these observations, it is worth investigating the ability of the **YCZ-series** to inhibit BR biosynthesis in monocot plants, such as rice. To explore whether BR biosynthesis inhibitors can induce phenotypes similar to those of mutants that are deficient for BR biosynthesis in rice, we conducted a screen of a compound library of the **YCZ-series** and evaluated the phenotypes. In this study, we report the discovery of new compounds with the ability to induce a phenotype similar to BR deficiency in rice plants. 

## 2. Results and Discussion

The chemical structures of the compounds are listed in Table 1. In our assays, we used hydroponic methods to culture the rice seedlings and evaluate the biological activity of the analogues of **YCZ-series** [16]. The common phenotypes of BR deficient mutants of rice plants are characterised by different degrees of dwarfism with erect leaves [6]. We chose plant height as a factor for the evaluation of the biological activity of the test compounds on rice seedlings. Another assay method that is known to be very sensitive to the levels of BR in rice is based on the determination of the bending angle of the lamina joint of the rice seedlings. The rice lamina joint is a tissue that is found between the leaf blade (leaf lamina) and the leaf sheath that acts similar to a hinge to bend the leaf blade. The bending angle of the lamina joint is sensitive to BR [17]. Furthermore, BR biosynthesis-deficient mutants of rice plants exhibit erect leaves with small bending angles of the lamina joint [18,19,20]. Thus, determination of the bending angle of the lamina joint of chemically treated rice plants is a straightforward approach to speculate the effects of the compounds used in this study on BR biosynthesis inhibition.

**Table 1:** Biological effects of **YCZ-series** analogues on the growth of rice seedlings.

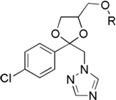		
No.	-R	(cm)
Cont	—	17.14 ± 1.08
1	Phenyl	9.65 ± 0.52
2	2-chlorophenyl	9.83 ± 0.41
3	3-chlorophenyl	15.21 ± 0.25
4	4-chlorophenyl	13.24 ± 0.48
5	2,3-dichlorophenyl	13.15 ± 0.50
6	2,4-dichlorophenyl	10.01 ± 0.43
7	2,5-dichlorophenyl	11.48 ± 0.53
8	2,6-dichlorophenyl	11.09 ± 0.36
9	3,4-dichlorophenyl	14.79 ± 0.42
10	3,5-dichlorophenyl	10.45 ± 0.75

### 2.1. Structure-Activity Relationships among the **YCZ-Series** with Regard to the Retardation of the Plant Height in Rice

To identify the chemical substituent on the phenoxy moiety that was responsible for the retardation of the growth of rice seedlings*,* various substituents were introduced onto the aromatic ring (compounds **1**-**10**). Compound **1**, which has no substituent on the phenyl ring, was used as a baseline reference for the structure-activity relationship. The concentrations of all of the test compounds were assigned to be 1 μM, and the plant height was measured. As shown in Table 1, the average plant height of the untreated control was approximately 17.14 ± 1.08 cm. Compound **1** exhibited an inhibitory effect on the growth of rice seedlings, resulting in a plant height of approximately 9.65 ± 0.52 cm. Analogue **2** contained a chlorine atom at position 2 of the phenyl ring and did not show a significant difference compared to compound **1** with regard to the retardation of the plant height. The rice seedlings treated with analogue **2** reached a height of approximately 9.83 ± 0.41 cm. Interestingly, moving the chlorine atom to position 3 or 4 (compound **3** and **4**) reduced the inhibitory activity, resulting in seedlings with a plant height of 15.21 ± 0.25 and 13.24 ± 0.48 cm, respectively. Analogues **5** through **10** contain two chlorine atoms with variations in the phenyl ring and exhibited different inhibitory potencies for the retardation of rice seedlings in our assay system. The data shown in Table 1 suggest that the introduction of the mono substituent of the chlorine atom at the *meta* position of the phenyl ring (compound **3**, **5**, **9**) may have a negative effect on the inhibitory activity of this synthetic series with regard to the retardation of plant height. Compound **1**, which has no chlorine substituent on the phenyl ring, is the most potent inhibitor. 

**Figure 2:**
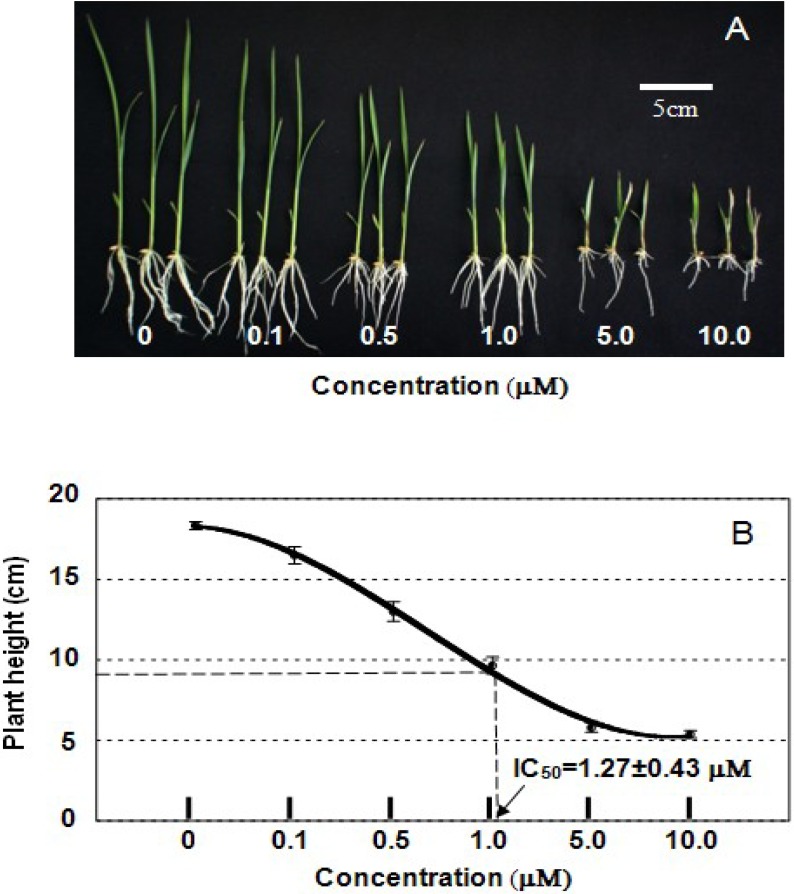
Effects of compound 1 on the height of rice seedlings. (**A**) The plants were treated with compound 1 at the specified concentration (0–10 μM) and grown under the conditions described in the experimental section for 10 days; (**B**) Average plant height of seedlings 10 days after treatment (*n =* 8). Error bars represent SD. The IC_50_ value of compound 1 for the inhibition of plant height was calculated by defining the plant height of untreated seedlings as 0% inhibition. The experiments were performed at least in duplicate to establish the repeatability.

To further determine the effect of compound **1** on the plant height in the rice seedlings, we treated rice seedlings with a different concentration of compound **1**. As shown in Figure 2A, the retardation of the growth of the rice seedlings treated with compound **1** was dose-dependent, and the IC_50_ value was approximately 1.27 ± 0.43 μM (Figure 2B). As the compounds in the **YCZ-series** are selective BR biosynthesis inhibitors that target the CYP90s in Arabidopsis [12], the data obtained here suggest that the ability of compound **1** to induce the dwarf phenotype in rice seedlings is likely due to the inhibition of BR biosynthesis in rice plants. 

### 2.2. Effect of the YCZ-series on the Bending Angle of the Lamina Joint of the Rice

**Figure 3:**
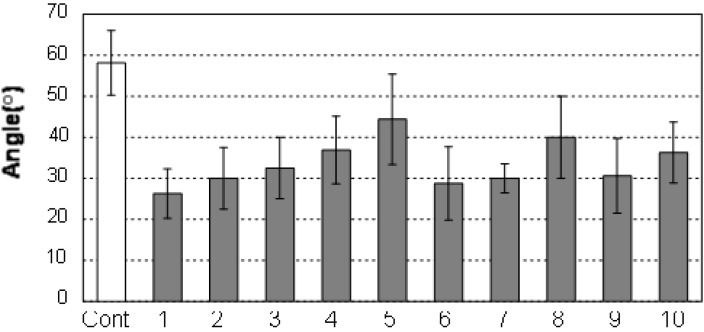
Effects of the YCZ-series on the lamina joint bending angle in rice seedlings. The test compounds were dissolved in the culture medium at a final concentration of 1 μM. The rice seedlings were growth under the conditions described in the experimental section. The lamina joint bending angle of 5-day-old rice seedlings was measured according to a method that has been previously described [17]. The data are the means SD of the results from eight plants. All of the experiments were performed at least in duplicate to establish the repeatability.

To further confirm the inhibitory effects of the **YCZ-series** on BR biosynthesis in rice seedlings, the small bending angle of the lamina joint, which is specifically affected in BR-deficient rice plants, was measured. The test compounds were added to the culture medium for a final concentration of 1 μM. We found that the chemical-treated rice seedlings exhibited different characteristics with regard to the bending angle of the lamina joint of rice plants. As shown in Figure 3, the bending angle of the lamina joint of the untreated control was approximately 58.1 ± 7.9 degrees, whereas the bending angle of the lamina joint in the chemical-treated rice plants was reduced to different degrees. With the exception of compounds **3** and **9**, the ability of the treatment to reduce the bending angle of the lamina joint was correlated with the reduced plant height of the rice seedlings. Among the test compounds, compound **1** was the most potent inhibitor for reducing the bending angle of the lamina joint, resulting in a bending angle of approximately 26.3 ± 6.0 degrees. 

**Figure 4:**
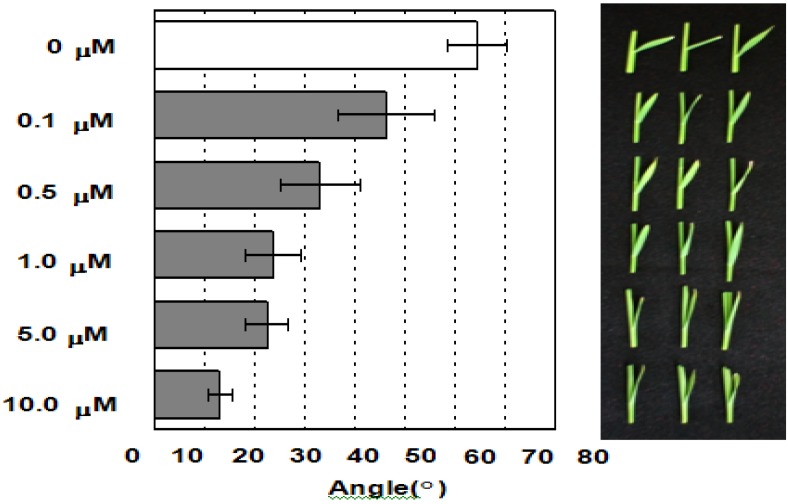
Effects of compound **1** on the lamina joint bending angle in rice seedlings. The average bending angle of the lamina joint was measured (*n =* 8). Error bars represent SD. The IC_50_ value of compound **1** for the inhibition of the bending angle of the lamina joint was calculated by defining the bending angle of the lamina joint of untreated seedlings as 0% inhibition. The experiments were performed at least in duplicate to establish the repeatability.

Next, we determined the effects of the concentration of compound 1 on the bending angle of the lamina joint. As shown in Figure 4, the bending angle of the lamina joint of the control was approximately 64.4 ± 5.8 degrees. In the presence of 0.1 μM of compound **1** in the culture medium, the bending angle decreased by 18.1 degrees. The bending angle decreased with an increase in the concentration of compound **1** in a dose-dependent manner. At a concentration of 10 μM of compound **1**, the bending angle was reduced to 13.1 ± 2.4 degrees. This result clearly indicates that compound **1** induced a characteristic phenotype of erect leaves, similar to that observed in BR biosynthesis-deficient mutant rice. 

The test compounds were dissolved in culture medium at a final concentration of 1 μM. The rice seedlings were grown under the conditions described in the experimental section. The plant heights of 10-day-old rice seedlings were measured. The data are the means ± standard deviation (SD) of the results from the eight plants. All of the experiments were performed at least in duplicate to establish the repeatability.

## 3. Experimental Section

### 3.1. Chemicals

The BR biosynthesis inhibitors used in this study were synthesised by a method that has been described previously [10]. Stock solutions of the test compounds were dissolved in DMSO at a concentration of 100 mM and stocked at −30 °C. The other reagents were of the highest grade and purchased from Wako, Pure Chemical Industries, Ltd. (Tokyo, Japan).

### 3.2. Plant Culture and Growth Conditions

Rice seeds (*Oryza sativa* L. cv. “Akitakomachi”) were sterilised in a 0.86 mM benomyl solution (a commercially available fungicide purchased from Sumitomo chemical garden products, Tokyo, Japan) for 24 h and washed with distilled water five times. The seeds were cold-treated (4 °C) for three days and then transferred to an incubator at 30 °C for two days to promote simultaneous germination. The germinating seeds were placed on gauze set on top of 200-mL disposable plastic cups (Sunplatec) and stimulated to germinate in distilled water for another three days. Subsequently, the distilled water was replaced with Hoagland’s solution containing several chemicals [16]. The growth conditions were set to 27 °C/25 °C in a 14 h light (Approximately 18,000 Lx)/10 h dark cycle with the humidity at 70%. The plant height of 10-day-old seedlings was measured after treatment with the chemicals. Average plant height was measured from 8 rice seedlings after chemical treatment or without chemical treatment for 10 days. The IC_50_ value of compound **1** for the inhibition of plant height was calculated by defining the plant height of untreated seedlings as 0% inhibition while the plant height of 0 cm was assigned to be 100% inhibition, and the IC_50_ value was calculated accordingly. All the experiments were done at least in duplicate to establish the repeatability.

### 3.3. Lamina Joint Bending Assay

The angle between the lamina and the second leaf sheath in 5-day-old seedlings after treatment with or without chemicals was measured. The second lamina joints were used for the lamina bending assay as described [17].

## 4. Conclusions

We screened a library of small-molecule inhibitors of BR biosynthesis to evaluate the biological activities of these compounds on rice plants based on the reduction of the plant height and their effects on the bending angle of the lamina joint of the rice plants. We observed that compound 1 exhibited a potent inhibitory effect on plant height in rice. The lamina joint bending assay indicated that these compounds induced erect leaves with a small bending angle at the lamina joint, which is similar to the phenotype of BR biosynthesis deficient mutants of rice. The biological activities of the compounds used in this study are in good agreement with the results of these two assays. Compound **1** is the most potent inhibitor among the analogues used in this study which inhibits BR biosynthesis in rice plants. 
